# Small Intestinal Adenocarcinoma Presenting With Internal High Echoes: A Case Report

**DOI:** 10.7759/cureus.88023

**Published:** 2025-07-15

**Authors:** Mami Yoshida, Shoji Oura

**Affiliations:** 1 Department of Surgery, Kishiwada Tokushukai Hospital, Kishiwada, JPN

**Keywords:** adenocarcinoma, internal high echoes, non-meckel’s diverticulum, small bowel tumors, small intestine

## Abstract

An 82-year-old female was referred to our hospital due to repeated vomiting. CT showed a heterogeneously enhanced mass, 50 mm in size, with cavitation in the lower abdomen. Ultrasound revealed a well-circumscribed polygonal mass with internal high echoes. Positron emission tomography (PET) of the target mass showed a maximal standardized uptake value of 18.6. Single-balloon endoscopy, however, failed to directly visualize the mass. Although serum CEA and CA19-9 levels were within normal ranges, sIL-2R was slightly elevated at 954 U/mL. Ultrasound findings, however, prompted us to operate on the target mass under the tentative diagnosis of ileal adenocarcinoma. The operation revealed that the tumor was located in the terminal ileal diverticulum and involved the surrounding small intestine. The diverticulum was not Meckel’s diverticulum and was located on the mesentery side. The mass did not pathologically contain any ectopic tissues and had atypical cells, mainly growing in a tubular fashion at the epithelium and in a cribriform fashion in the muscular propria. Immunostaining showed positivity for CK7 and CDX2, partial positivity for MUC5AC, and negativity for CK20, CD10, CD56, synaptophysin, and chromogranin. These pathological findings led to the diagnosis of ileal moderately-differentiated adenocarcinoma. The patient recovered uneventfully, was discharged on the 10th day after surgery, and was scheduled for outpatient follow-up without adjuvant chemotherapy given her advanced age. Diagnostic physicians should note that moderately- or well-differentiated adenocarcinomas have internal high echoes, which can be an important diagnostic clue for the differential diagnosis of small intestinal tumors.

## Introduction

Small intestinal tumors are rare malignancies, accounting for less than 5% of all gastrointestinal tract malignancies; they consist mainly of neuroendocrine tumors, adenocarcinomas [1], malignant lymphomas, and sarcomas, including gastrointestinal stromal tumors (GISTs) in order of their frequency [2]. In addition, the vast majority of cases manifest no symptoms until some kind of obstruction- or infiltration-induced symptoms appear. The advent of capsule endoscopy [3] and device-assisted enteroscopy [4] has enabled us to endoscopically observe small intestinal tumors in some cases. However, there are no standard image modalities that enable us to make a differential diagnosis of small bowel tumors. While ultrasound is useful for diagnosing various solid cancers, there is scarce research about the ultrasound diagnosis of small intestinal tumors, as the presence of air in the digestive tract often makes ultrasound imaging difficult.

It is well known that local stimuli, including infection, can cause several cancers. Hepatitis B/C [5,6] and human papillomavirus [7] infection are highly associated with the increased risk of hepatocellular carcinoma and uterine cervix cancer, respectively. The low incidence of small bowel cancer may be attributed to the lower susceptibility of the small intestine to irritation than the stomach and the large intestine. Meckel's diverticulum [8], therefore, is the part of the small intestine most susceptible to local irritation and is the most common site for the development of small intestinal cancer in the jejunum and ileum. We report a case of a small bowel tumor developed in the non-Meckel’s diverticulum, which was determined to be small intestinal cancer based on preoperative imaging, especially internal echo, findings.

## Case presentation

**Figure 1:**
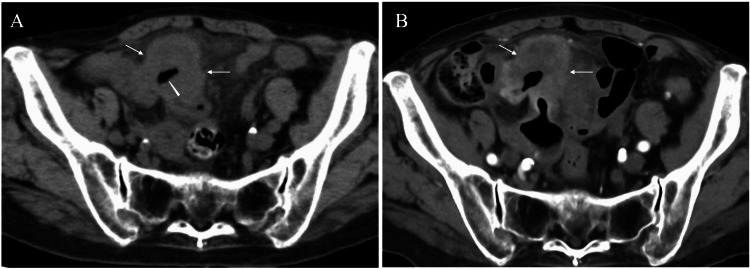
CT findings CT images showed an oval mass (arrows) with cavitation (arrowhead) in the lower abdomen (A) and a weak enhancement of the mass (arrows), especially in the peripheral areas of the mass (B) CT: computed tomography

**Figure 2:**
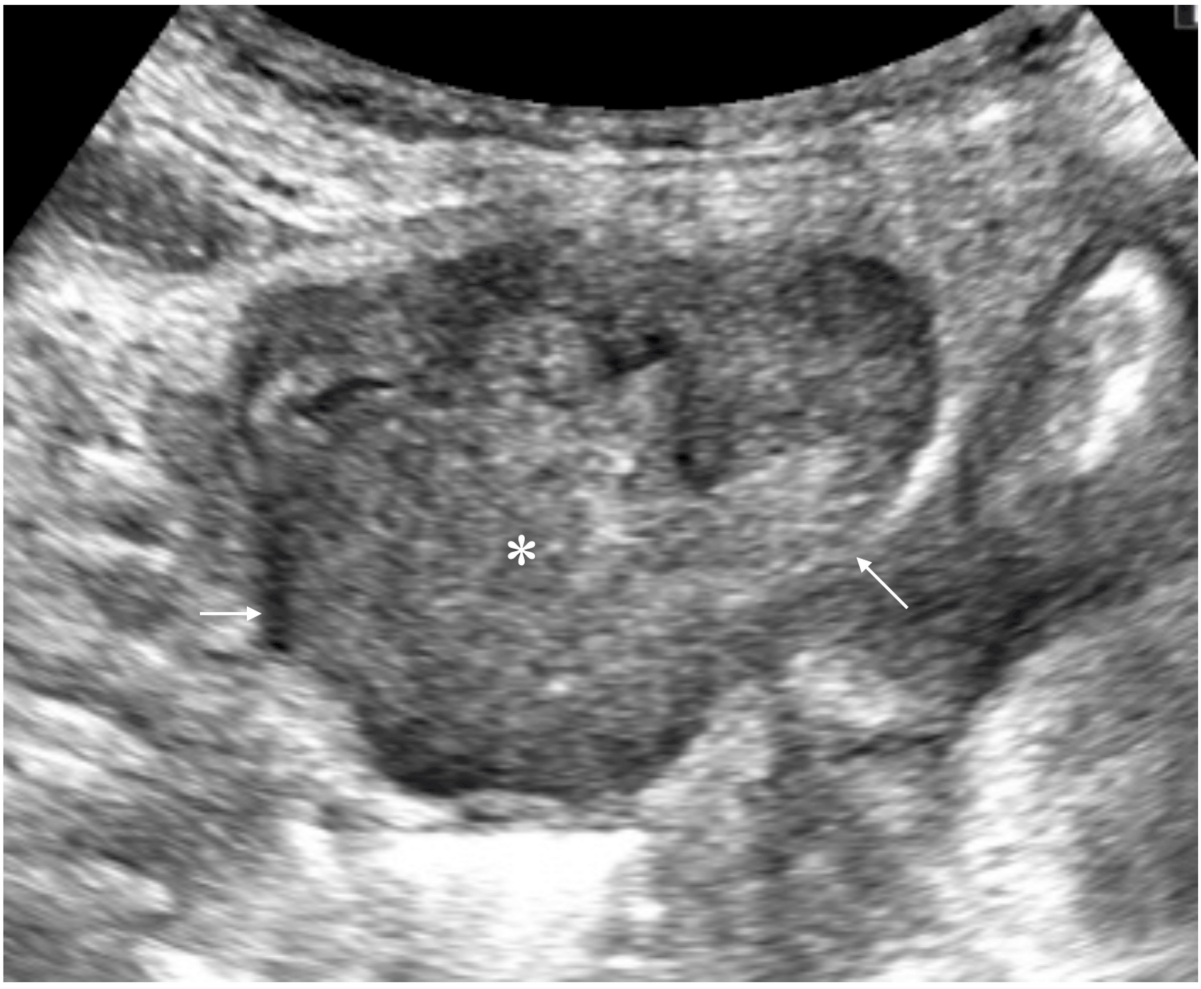
Ultrasound findings Ultrasound showed a well-circumscribed polygonal mass (arrows) with internal high echoes (asterisk)

An 82-year-old female was referred to our hospital due to repeated vomiting. CT showed a heterogeneously enhanced mass, 50 mm in size, with cavitation in the lower abdomen (Figure 1). The Ultrasound showed a well-circumscribed polygonal mass with internal high echoes (Figure 2).

**Figure 3:**
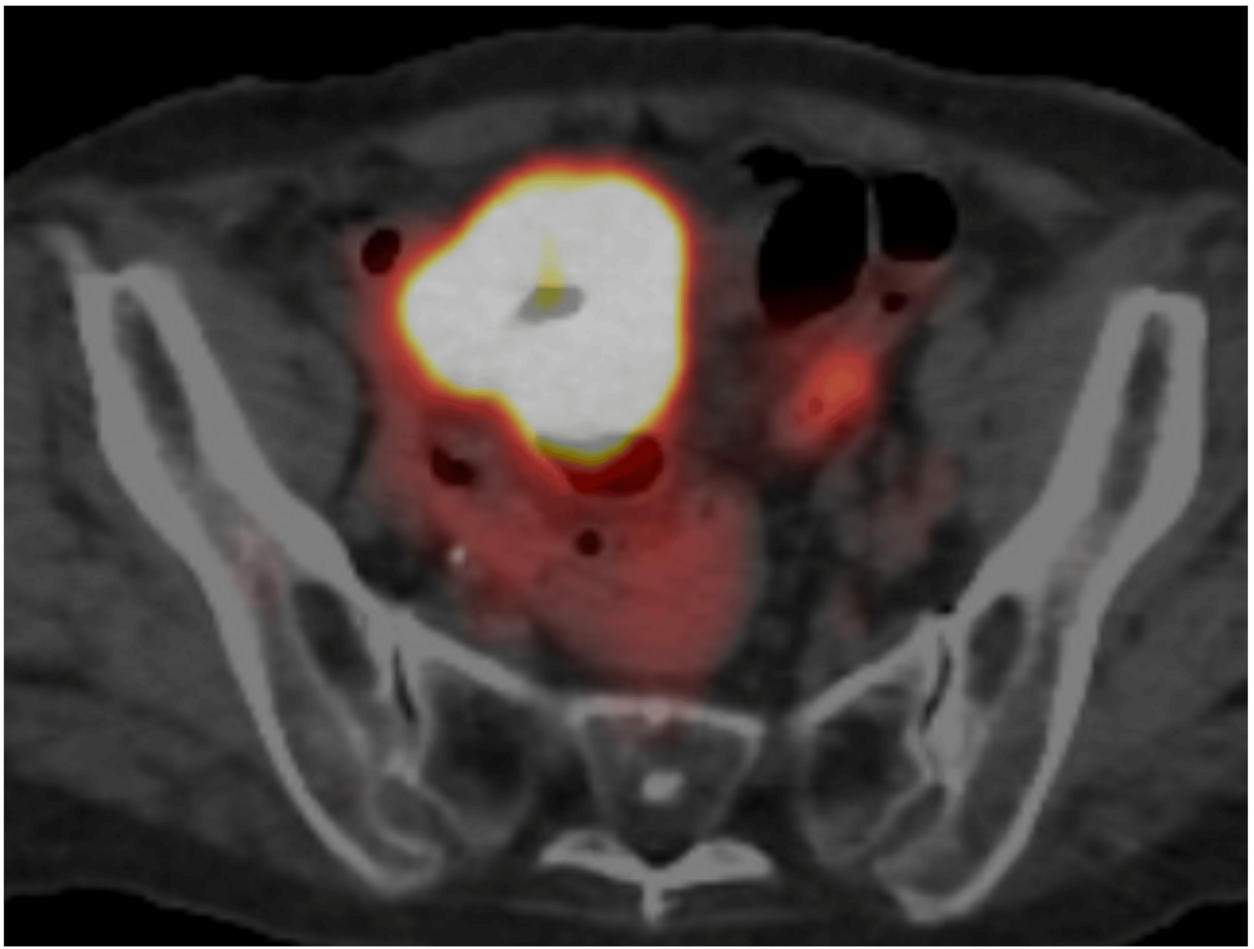
PET/CT findings PET/CT showed avid fluorodeoxyglucose uptake in the mass, especially in the peripheral areas of the mass PET/CT: positron emission tomography/computed tomography

Colonoscopy revealed a colonic diverticulum, but could not identify any neoplastic lesions. Under the tentative diagnosis of a small bowel tumor, the patient underwent single-balloon endoscopy, which unfortunately only resulted in a very limited level of examination of the small intestine and could not identify any small intestinal tumors. Positron emission tomography (PET) of the target mass showed a maximal standardized uptake value of 18.6 (Figure 3).

**Figure 4:**
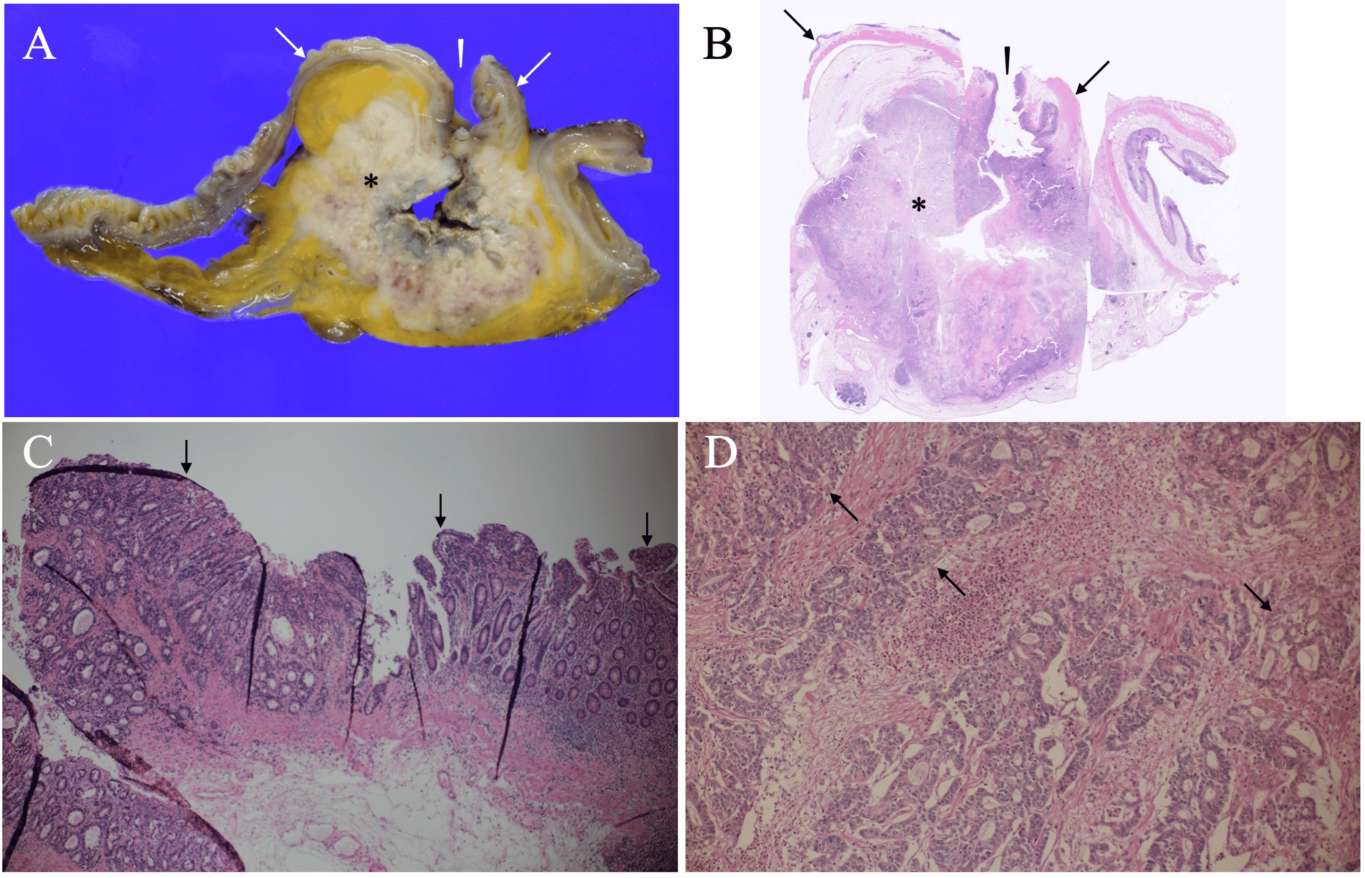
Pathological findings A. Cut surface of the mass showed normal ileal mucosae (arrows) and a polygonal tumor (asterisk) in the ileal diverticulum (arrowhead). B. Low magnified view showed that the mass (asterisk) was covered with normal ileal mucosae (arrows) and had cancer spread to the ileal diverticulum orifice (arrowhead) (H.E.x20). C. Mucosae of the diverticulum (arrows) had adenocarcinoma cells growing mainly in a tubular fashion (H.E.x100). D. Magnified view showed invasive adenocarcinoma cells growing mainly in a cribriform fashion (arrows) in the muscularis propria (H.E.x200)

Although serum CEA and CA19-9 levels were within normal ranges, sIL-2R showed a slightly elevated level of 954 U/mL (reference range: 122-496 U/mL). These findings, especially not the sIL-2R level but the ultrasound findings, made us suspect the target mass to be a small intestinal cancer. Therefore, after the anemia treatment with blood transfusion, the patient underwent surgery for the target mass. The operation revealed that the tumor was located in the terminal ileal diverticulum at the mesentery side and involved the surrounding small intestine. The lack of lymphadenopathy in the ileal mesentery around the tumor made us resect the tumor with 10 cm safety margins both for the oral and anal sides and with lymph node dissection up to the lymph nodes at the bifurcation of the ileocolic artery to the small intestine. Postoperative pathological study showed that the mass had no lymph node metastasis, no ectopic tissues, and atypical cells mainly growing in a tubular fashion at the epithelium and in a cribriform fashion in the muscularis propria, leading to the diagnosis of moderately differentiated adenocarcinoma (Figure 4).

Immunostaining of the tumor showed positivity for CK7 and CDX2, partial positivity for MUC5AC, and negativity for CK20, CD10, CD56, synaptophysin, and chromogranin, highly aligning with the diagnosis of ileal adenocarcinoma. The patient recovered uneventfully, was discharged on the 10th day after operation, and was scheduled for outpatient follow-up without adjuvant chemotherapy due to her advanced age.

## Discussion

Most neuroendocrine tumors in the small bowel occur in the duodenum, particularly at or around the papilla [9]. While neuroendocrine tumors can also develop in areas beyond the duodenum, the vast majority of them occur near the terminal ileum. Compared to duodenal neuroendocrine tumors, those in the jejunum and ileum are mostly non-functioning tumors and are overwhelmingly discovered with some symptoms due to tumor growth or are incidentally detected on some kind of image evaluation. In our case, the initial symptom was repeated vomiting due to the ileal tumor, which grew in the mesentery side diverticulum near the terminal ileum.

Neuroendocrine tumors are broadly classified into well-differentiated and poorly differentiated subtypes. Differentiation degrees of gastric and colorectal cancers are mainly defined by the type and degree of tubule-forming structures, while those of neuroendocrine tumors are determined by the mitotic and the Ki-67 labelling indices. In other words, even well-differentiated neuroendocrine tumors generally have no tubule-forming structures. Neuroendocrine tumors, therefore, generally have internal low echoes on ultrasound, even in well-differentiated cases. Similarly, malignant lymphomas [10] and GISTs [11] have tubule-forming structures in very rare cases only. Tubule-forming structures, therefore, may well aid in the differential diagnosis between small bowel cancers, especially well-to moderately-differentiated adenocarcinomas, and other disorders.

PET showed an elevated standardized maximal uptake value of 18.6 in our case, which implied the highly progressive characteristics of the tumor but did not contribute to the differential diagnosis of small intestinal tumors. CT clarified the cavitation of the mass, which suggested that the mass developed from the ileal mucosa or was at least located near the ileal mucosa. In this case, the cavitation actually reflected the diverticular lumen.

Reflection and backscattering of ultrasound waves generate the mass shapes and the internal echoes, respectively. Ultrasound reflection needs interfaces, i.e., much larger than the ultrasound wavelength, to depict mass borders that form the mass shape. Ultrasound wave backscattering occurs when ultrasound waves hit backscatter-inducing structures, including some kind of micro voids [12], or collide with scattering bodies, i.e., smaller than the ultrasound wave length, with different acoustic impedance from that of adjacent pathological components, both generating internal high echoes of masses. Therefore, when tumors have either tubule-forming structures or some pathological components with different acoustic impedances from those of malignant cells, such as fat cells, they show high internal echoes. Conversely, it is well known that malignant lymphomas generally have very low internal echoes due to the striking similarity of acoustic impedance among mass-constituting lymphoma cells [13]. GISTs similarly have internal low echoes at a very high rate. Although no studies have reported the ultrasound findings of neuroendocrine tumors, their pathological characteristics can make us speculate that they have low internal echoes. Diagnostic physicians, therefore, should keep in mind that moderate-to-well-differentiated adenocarcinomas have high internal echoes, which is very useful in the differential diagnosis of small intestinal tumors.

## Conclusions

Diagnostic physicians should engage in a differential diagnosis of small bowel tumors by keeping in mind various images that correspond to the pathological features of neuroendocrine tumors, small intestinal adenocarcinomas, malignant lymphomas, and sarcomas, including GISTs. In other words, it should be noted that, of the four main tumorous disorders in the small intestine, neuroendocrine tumors, malignant lymphomas, sarcomas, including GISTS, and poorly differentiated adenocarcinomas generally have low internal echoes. Diagnostic physicians should note that internal high echoes of masses can provide an important clue in the diagnosis of small bowel tumors. Further research is required to explore the strong correlation between internal high echoes and moderately-/well-differentiated small intestinal adenocarcinomas.
